# Emerging technologies for monitoring breast cancer response to neoadjuvant chemotherapy: a systematic scoping review

**DOI:** 10.1016/j.breast.2025.104663

**Published:** 2025-12-01

**Authors:** Georgia C. Wright, Luke T. Glover, Thomas J.E. Hubbard

**Affiliations:** a Royal Devon University Hospitals NHS Foundation Trust, Barrack Road, Devon, Exeter, EX2 5DW, UK; b University Hospitals Plymouth NHS Trust, Derriford Road, Crownhill, Devon, Plymouth, PL6 8DH, UK; c University of Exeter Medical School, St Luke's Campus, Heavitree Road, Exeter, EX1 2LU, UK

**Keywords:** Neoadjuvant chemotherapy, Pathological complete response, Technology readiness level, Novel technology, Circulating nucleic acid, Elastography, Diffuse optical imaging

## Abstract

**Introduction:**

Neoadjuvant chemotherapy (NACT) is increasingly used in early breast cancer treatment and responses are highly variable. Accurate monitoring of tumour response is crucial for enabling precise surgical de-escalation, yet current methods are inadequate.

This systematic scoping review explores emerging technologies for predicting, monitoring, and diagnosing pre-operative breast cancer response to NACT.

**Methods:**

A search of Embase, Medline, PubMed, and Cochrane databases was conducted until January 26, 2024. Studies investigating ability to detect tumour response during or after NACT, and prior to surgery, were included, and placed into 12 technology categories. Those investigating novel technologies were further categorised by Technology Readiness Level.

**Results:**

From 2497 studies, 1329 met the inclusion criteria. 479/1329 (36 %) investigated conventional imaging; 19 % (253) investigated MRI, 5 % (64) mammography/ultrasound, 0.5 % (6) computed tomography, 0.4 % (5) digital breast tomosynthesis. Established technologies included gene panels (134/1329; 10 %) and post treatment core biopsy 35/1329; (3 %). 107/1329 (8 %) of studies developed nomograms based on routine clinical investigations, and 493/1329 (37 %) correlated established biomarkers e.g. Ki-67 with pathological response.

81 studies (6 %) addressed novel technologies, such as circulating nucleic acids (49/81), diffuse optical imaging (17/81), and elastography (15/81). A Technology Readiness Assessment revealed that all were between Technology Readiness Level 2 (Invention and Research) and 6 (Large Scale).

**Conclusions:**

The majority of current research activity focuses on optimising existing technologies which may never provide the step change in diagnostic accuracy required to advance surgical de-escalation. Research activity should be focused on identifying effective novel technologies and driving translation into the clinical environment.

## Highlights

• Predicting pathological complete response to neoadjuvant chemotherapy is an active field.
• Abundant research on established technologies, sparse on novel technologies.
• Circulating nucleic acids, elastography and diffuse optical imaging show promise.
• Technology Readiness Level can be used to compare development of novel technologies.

## 1 Introduction

Breast cancer is the most common cancer to affect women in the world, accounting for 15 % of all cancer diagnoses and an incidence of 55,000 cases per year in the UK [1]. The traditional paradigm of early breast cancer treatment is primary surgery, with subsequent relevant adjuvant therapies administered according to the final surgical specimen pathology. Neoadjuvant chemotherapy (NACT)– where systemic chemotherapy treatment precedes surgical resection – is an established treatment for inoperable or advanced breast cancer [2], and is being increasingly used in early breast cancer [3,4]. NACT has a number of potential advantages; it may downsize tumours to facilitate Breast Conserving Surgery (BCS) in patients that otherwise would have undergone mastectomy, or it may downstage pathologically diseased axillary nodal disease to facilitate sentinel node biopsy/Targeted Axillary Dissection in patients that otherwise would have undergone axillary clearance. It can also facilitate *in vivo* assessment of the tumour response to chemotherapy agents, allowing for a change in treatment, or tailored adjuvant treatments and prognostication [5,6]. With the rapid advances in modern chemotherapy agents, and the desire to de-escalate surgical interventions, the indications for NACT are expanding [7] and it is likely that the proportion of patients with breast cancer undergoing NACT will increase from its current median of 10 % (with a range of 5–60 %) in a recent UK survey [4].

Tumours in patients undergoing NACT can have a range of responses to systemic treatment [5]. The tumour may progress despite systemic treatments (if identified leading to a change in management), remain stable, have a partial response (with residual tumour after treatment) or have a complete pathological response (pCR) where there is no residual invasive tumour detected on surgical histology after excision. It is currently difficult to predict how tumours will respond to NACT. The most common tumour characteristic used to determine whether NACT should be instituted is tumour biology (e.g. receptor status), as this is correlated with therapeutic targets and/or chemosensitivity [8]. For example, Human Epidermal Receptor – 2 (HER2) positive, oEstrogen Receptor (ER) negative tumours can achieve a pCR rate of up to 90 % [9], while ER positive HER2 negative tumours have pCR rates of less than 20 % [8]. Such markers are often used along with the tumour size and/or lymph node status, as this dictates if there are potential gains to be had from surgical de-escalation [6,10].

The final response to NACT is currently determined after surgical excision of the area of breast tissue where the cancer was initially biopsied and diagnosed (usually identified by a marker clip that is deployed into the area at the initial biopsy) and a histopathological diagnosis of the excised surgical specimen is made. A pathologically complete response (pCR) can have a number of definitions. All definitions agree that there should be complete eradication of invasive cancer cells in the breast tissue, but some allow for persistent *in situ* disease, and/or residual disease in axillary lymph nodes [11]. A pooled analysis demonstrated that patients with pCR where this was defined as the complete eradication of invasive and *in situ* disease in both the breast and axilla had the lowest disease free survival hazard ratios [12]. The CTneoBC meta-analysis demonstrated that a pCR as defined by no residual *invasive* disease in either breast or axilla was associated with an improved Event Free Survival, but could not validate pCR as a surrogate endpoint for improved EFS and OS [8]. Subsequent studies have demonstrated that patients with a pCR have an improved Overall Survival compared with those that did not, but only in some sub-types [13,14].

The presence of residual disease in the surgically excised specimen can prompt the recommendation of further adjuvant chemotherapy regimes based on recent trials such as the KATHERINE trial [15] and CREATE-X trial [16], allowing for treatments to be tailored to tumour chemo-responsiveness.

### 1.1 Clinical importance of monitoring tumour response to NACT

Assessment of tumour response to NACT allows consequent alteration of treatment course prior to surgery (e.g. a switch in chemotherapy or cessation to prevent unnecessary toxicity if initial regimes are futile) or change in operative management (e.g. deciding on mastectomy vs Breast Conserving Surgery). Any of these changes can only be made with accurate knowledge of the tumour response prior to surgery.

One challenge in the assessment of response to NACT lies in the significance of breast calcifications. Breast calcifications are associated commonly with ductal carcinoma *in situ* (DCIS), but also with invasive disease, and are seen in 20–50 % of patients before instituting NACT [[9], [10], [11], [12], [13], [14], [15], [16], [17]]. Calcification may result from tumour necrosis [20], or be induced by low pH within the tumour microenvironment. It is likely that calcifications have an association with cancer rather than being intrinsically malignant [21]. Calcification biochemistry has been noted to be associated with pathological diagnosis [22,23] and tumour grade. An insight into the complex relationship of breast cancer and calcification is exemplified by the range of responses in breast calcification seen in response to NACT – they can increase, decrease or remain stable, and this is independent of the tumour response [[17], [18], [19], [20],^24^]. Due to the lack of understanding of the oncological significance of breast calcification after NACT, surgical excision of residual calcifications is recommended [18,25] which may reduce BCS rates, or even exclude patients from NACT if there are widespread calcifications, and this is a limitation to surgical de-escalation after NACT currently.

The unifying clinical issue is that current methods to monitor the tumour response of patients undergoing NACT are inadequate. A recent UK national audit of practice has demonstrated the surgical consequences of the current inability to pre-operatively diagnose a pCR; 45 % of patients underwent resection of the pre-treatment tumour footprint, and 48 % of those who underwent an axillary node clearance had pathologically negative nodes [6,26].

**Fig. 1:**
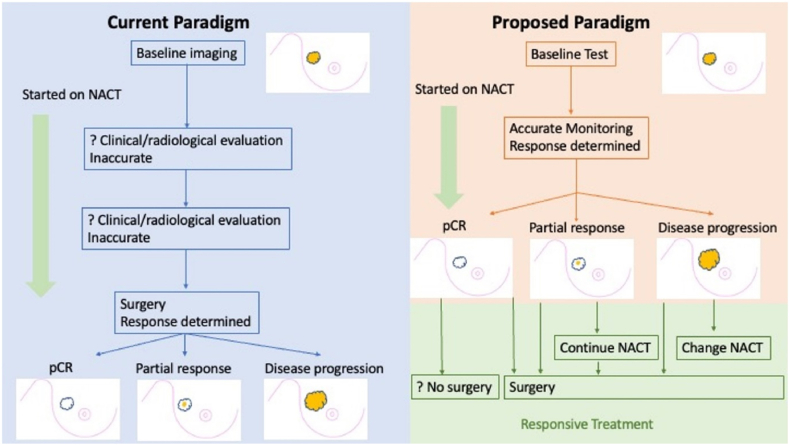
Example of how accurate diagnosis of tumour response to NACT could alter treatment paradigm. NACT – Neoadjuvant chemotherapy, pCR – pathological complete response.

If NACT could be accurately monitored, this would change the current paradigm of breast cancer management (see Fig. 1). Accurate determination of poor response could enable switching of chemotherapy regimens, or earlier institution of surgery. Conversely, if partial tumour response or a pCR were accurately identified, surgical de-escalation could be instituted more appropriately. There are a number of studies pursuing the ultimate surgical de-escalation with omission of surgery [27], initially investigating the accuracy of systematic and extensive image guided tumour bed biopsies to predict a pCR in the subsequently surgically resected specimen, with false negative rates ranging from 5 to 40 % [[28], [29], [30], [31]]. MD Anderson demonstrated an acceptably low false negative rate in the preliminary biopsy trial [31], and have initial reports of a trial in a small number of biopsy-diagnosed exceptional responders who have had complete omission of surgery, with promising early results [32].

Accurate de-escalation, even to the point of omission, of surgery is likely to only be achieved outside of the context of a trial if there is accurate *in vivo* monitoring of tumour response to NACT. This has been identified as a top priority in a recent research gap analysis [33] and priority setting for breast surgery research [34].

### 1.2 Current methods of monitoring response to NACT

Current guidelines around use of imaging to monitor the response to NACT are not didactic, demonstrating that no single imaging technique is accurate enough to significantly alter management and therefore be accepted as a standard practice of care. NICE guidelines do not mandate any imaging during the course of NACT [10], and recent UK based consensus guidelines suggest imaging at the end of treatment either in the form of MRI if one was performed at baseline, or mammogram (MMG) with or without ultrasound (US) [6], which are similar to current American Society of Clinical Oncology (ASCO) guidelines [7].

The accuracy of these established imaging techniques (MMG, US + MRI) in monitoring response to NACT has been extensively investigated. Although accuracy varies between cancer subtypes, neither mammogram, ultrasound, nor MRI is sufficiently accurate currently to replace surgical excision in the diagnosis of pCR [83]. A summary of the reported accuracies of established techniques is presented in Table 1.

**Table 1:** Summary table of the mode of assessment of the breast tumour during NACT ability to predict a pCR.

Mode of assessment	PPV	NPV	Overall Accuracy	Ref
Clinical Examination	75–87 %	28–41 %	54 %–75 %	[[35], [36], [37], [38]]
Mammogram	54–82 %	30 %-86 %	71 %–84 %	[35,39]
US	68–85 %	33 %–85 %	78 %–81 %	[36,39,40]
MRI	63–99 %	44 %-84 %	70 %–86 %	[36,41,42]

As potentially the most accurate modality, MRI ability to predict a pCR has been the subject of both a systematic review [43], reporting non-pooled study level MRI accuracy, and a meta-analysis [44]. Both of these studies demonstrated that MRI both over- and underestimates tumour size after NACT with a wide range of correlation coefficients (0.21–0.92) between MRI and pathological tumour size, concluding that MRI produced clinically significant measurement errors. An up to date meta-analysis demonstrates that tumour sub-type affects the accuracy of MRI findings and may account for the wide differences [45]. The difficulty in accurately predicting the pathological response may account for the consistently high mastectomy rate in patients undergoing NACT, despite being in an era of high complete pathological complete response [[79], (a), (b), [80], [81]].

### 1.3 Aims of this scoping review

A comprehensive and systematic review of the literature pertaining to established radiological techniques in routine practice is discussed in other reviews [46] and is not the aim of this study, which proposes to evaluate which novel techniques are being investigated. There are few truly new methods of monitoring breast tumour response to NACT and predicting a pCR. The majority of research is focused on optimising the existing major imaging modalities of mammogram (MMG), ultrasound (US) and magnetic resonance imaging (MRI). However, given their lack of biochemical sensitivity as purely imaging techniques it may be that these modalities have a ceiling of diagnostic accuracy.

A scoping review was the most appropriate method as these emerging techniques are at various stages of development towards clinical application, with most not having undergone rigorous clinical testing, and therefore quantitative analysis (such as meta-analysis) is not possible.

The aim of this scoping review is to provide an overview of the emerging technologies that are currently in development towards monitoring breast cancer response to NACT, to explore the breadth and extent of the literature, and summarise the evidence. Ultimately this paper's goal is to identify those fields that hold the most potential in resolving the clinical question and informing future research.

### 1.4 Review question

What novel technologies are being developed to accurately monitor breast cancer response to NACT, and what is their stage of development?

## 2 Methods

### 2.1 Eligibility criteria

#### 2.1.1 Inclusion criteria

Studies using human tissue or human subjects investigating the ability to detect or predict changes in breast cancer after/during a course of NACT were included. Technologies only investigated in animal models were included. Technologies that only have reports using cell cultures were excluded.

Monitoring response to NACT differed according to the technique – Any technique that attempted to predict the final pathological diagnosis by size of tumour or completeness of response (e.g. pCR) was included.

This scoping review will consider both experimental and quasi-experimental study designs including randomised controlled trials, non-randomised controlled trials, before and after studies. All analytical observational studies both prospective and retrospective and conference abstracts are included.

#### 2.1.2 Exclusion criteria

Studies investigating the prognostic rather than predictive ability of a technology, i.e. with survival as an endpoint rather than pathological response, were excluded.

Qualitative studies and reviews were excluded.

### 2.2 Search strategy

The scoping review was conducted in accordance with the JBI methodology for scoping reviews and with reference to the JBI template [47]. The PRISMA-ScR checklist is included as Supplementary Fig. 1.

The search strategy aimed to locate both published and unpublished studies. An initial limited search was performed on MEDLINE to identify relevant articles, and the text words in the titles and abstracts used to develop a full search strategy.

Studies published in the English language were included, from 2002 onwards. This was felt to be appropriate, as any technology not in routine clinical use with no reports in the last 20 years are unlikely to be of clinical relevance.

A search of the biomedical literature up to January 2024 was performed. The databases searched were EMBASE and Medline via OVID, the Cochrane library, and Pubmed. The full search strategy used in the last search on 26/1/24 is elaborated in the supplementary material.

**Fig. 2:**
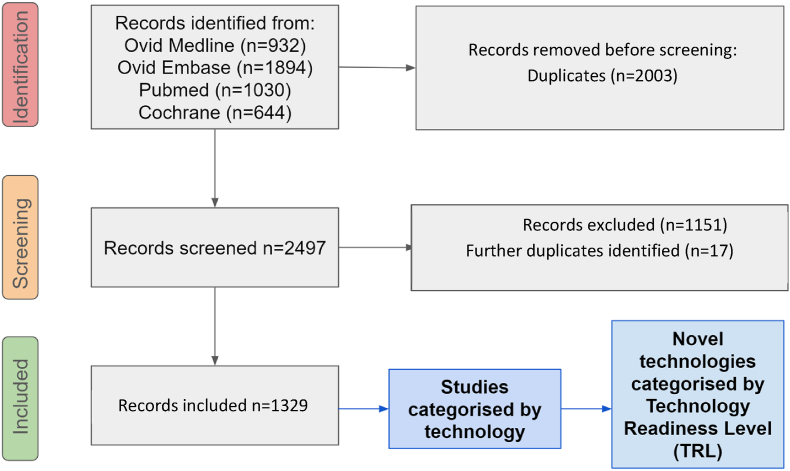
Modified PRISMA flow diagram.

Following the search, all identified citations were collated and uploaded to Endnote X9 (Clarivate Analytics, PA, USA). Duplicates were removed using the process described by Bramer et al., 2016 [48]. Titles and abstracts were screened by two independent reviewers for assessment against the inclusion criteria; a concordance check was performed using a randomly selected sample of 100 studies, following which inclusion and exclusion criteria were refined, 12 technology categories were defined, and the remainder of the studies were screened. Any disagreements arising between the reviewers at stage of the selection process was arbitrated by discussion and through arbitration of a third reviewer. (see Fig. 2).

### 2.3 Analysis

Included studies were placed into one of 12 technology categories, defined by discussion between the authors after the concordance check.

To identify novel studies the recent UK government definition of medical technology innovation was used [49]. Included technologies were those that were novel and innovative or disruptive.

Technologies of incremental improvement, copycat devices or those of discovery and invention were excluded (as not defined as innovative). Those technologies with research focused on incremental improvement (i.e. established techniques with optimisation, such as MRI) were also excluded as they are innovative, but not novel. Those studies deemed to be novel in this context were further categorised by technology readiness level (TRL).

The TRLs are a standardised framework used to describe the developmental stage of a given technology, ranging from TRL 1, ‘basic principles observed’, to TRL 9, ‘actual system proven in operational environment’. They were originally developed by NASA to evaluate space technologies, but have since been widely adopted across many fields including biotechnology and diagnostics. The TRL definitions used in this study were adapted from the US Biomedical Advanced Research and Development Authority [50].

## 3 Results

### 3.1 Study population

The study selection process is displayed in Fig. 2. 4500 articles in total were retrieved from the four databases searched, and after duplicates were systematically identified and deleted [48] a library of 2497 citations remained. These were screened by title and abstract and either excluded (1151/2497, 46 %), identified as further duplicates (17/2496, <1 %) or sorted into categories by the type of technology being investigated. This left 1329 studies for inclusion. Of note, 632 (47.5 %) were published in or since 2020, showing a rapid increase in publications in this field.

**Fig. 3:**
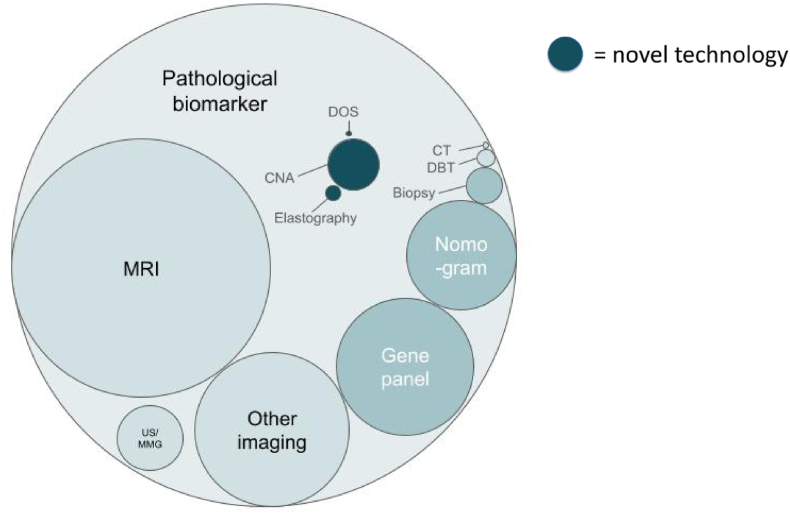
Proportional area chart of technology categories. DOS, Diffuse optical spectroscopy; DBT, Digital breast tomosynthesis; CNA, Circulating nucleic acids.

**Fig. 4:**
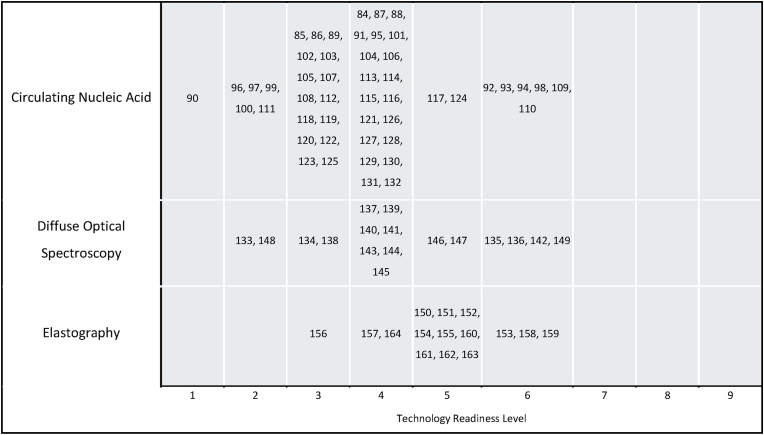
Distribution of TRLs in each novel technology category. Numbers in the main body of the figure correspond to this paper's references below.

The largest category of included studies concerned pathological biomarkers (493/1329, 37 %), followed by MRI (253/1329, 19 %), ‘Other imaging’ i.e. imaging processes besides MRI, CT, USS or MMG (151/1329, 11 %), and gene panels (134/1329, 10 %). Table 2 and Fig. 3 display the remaining categories and their proportional representation (see Fig. 4).

**Table 2:** Technology categories represented.

	Number of studies (% of included)
**Established imaging**	
CT	6 (0.5)
MRI	253 (19)
USS/MMG	64 (5)
Digital breast tomosynthesis	5 (0.4)
Other imaging	151 (11)
**Established biotechnologies**	
Biopsy	35 (3)
Nomogram	107 (8)
Gene panel	134 (10)
Biomarker	493 (37)
**Novel technologies**	
Circulating nucleic acids	49 (4)
Diffuse optical spectroscopy	17 (1)
Elastography	15 (1)

### 3.2 Timing of measurements

The largest and most diverse category was pathological biomarkers, to include a variety of molecular targets such as hormone receptor status, Ki67, markers of inflammatory and/or immune response, and circulating tumour cells. The majority of studies in this category assessed the biomarker in question at baseline, prior to delivery of NACT (429/493, 87 %). Others measured the biomarker at a number of timepoints during the course of NACT (18/493, 4 %), and some measured the biomarker after NACT and immediately prior to surgery (46/493, 9 %). See Supplementary Fig. 1 for a summary of the biomarkers measured at interim timepoints and post-NACT.

### 3.3 Novel technologies

Three technology categories were felt to be novel in this context: diffuse optical spectroscopy, circulating nucleic acids, and elastography. These categories constituted a total of 81 studies (81/1329, 6 %), and were further categorised according to Technology Readiness Level (TRL) [2], [3], [4], [5], [6], [7], [8], [9], [10], [11], [12], [13], [14], [15], [16], [17], [18], [19], [20], [21], [22], [23], [24], [25], [26], [27], [28], [29], [30], [31], [32], [33], [34], [35], [36], [37], [38], [39], [40], [41], [42], [43], [44], [45], [46], [47], [48], [49], [50], [51], [52], [53], [54], [55], [56], [57], [58], [59], [60], [61], [62], [63], [64], [65], [66], [67], [68],[68], [69], [70], [71]. The technology category with the highest average TRL was elastography (n = 15, median TRL 5, mean TRL 4.93) followed by DOS (n = 17, median 4, mean 4.24) and CNA (n = 49, median 4, mean 3.71) (see Fig. 4).

As shown in Table 3, accuracy and/or area under the receiver operating characteristics curve (AUC) figures were only sporadically reported by studies pertaining to novel technologies. There was a wide range of figures given, but the median in each category is comparable or better than the accuracy thus far achieved by MRI [36,41,42].

**Table 3:** Accuracy and area under the receiver operating characteristics curve (AUC) of studies regarding novel technologies.

	Studies reporting AUC (percentage of total)	Median AUC (range)	Studies reporting accuracy	Median accuracy (range)
CNA (n = 50)	6 (12 %)	0.928 (0.79–0.961)	3 (6 %)	92 % (83–93.33 %)
DOS (n = 17)	6 (35 %)	0.859 (0.6–0.95)	0	n/a
Elastography (n = 15)	11 (73 %)	0.855 (0.75–0.96)	1 (0.07)	83 %

**Fig. 5:**
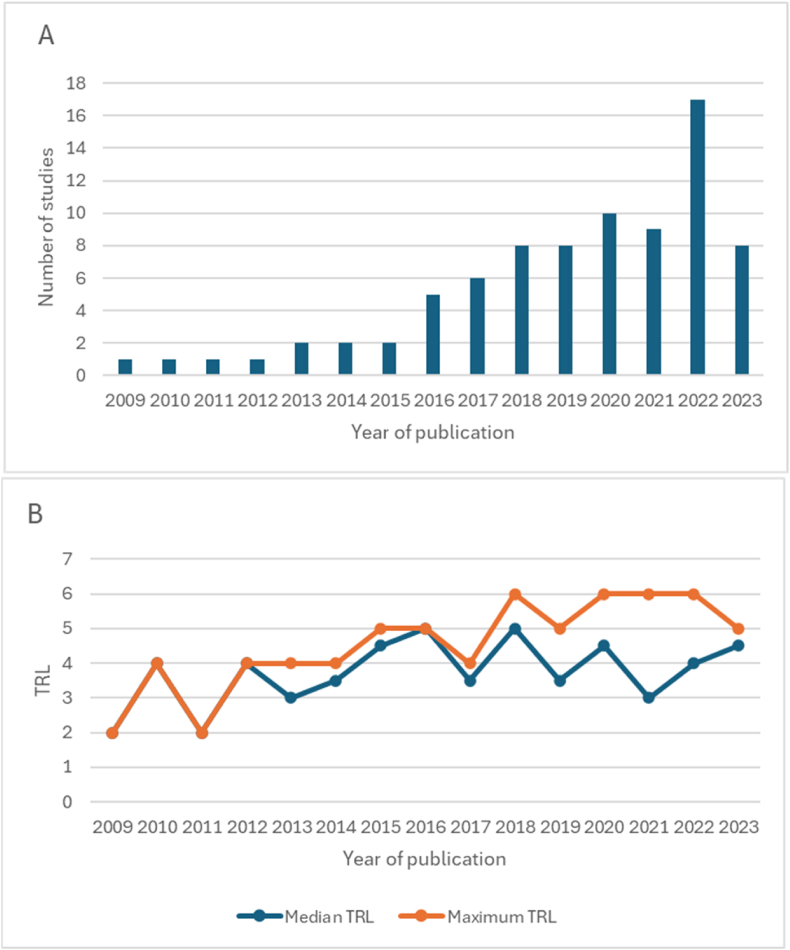
A) Total number of studies pertaining to novel technologies (circulating nucleic acids, direct optical spectroscopy, elastography) published each year B) Median and maximum TRL achieved each year by all studies pertaining to novel technologies.

Fig. 5 demonstrates that there has been accelerating activity in the three novel technology fields over the last two decades, as well as progression in the developmental stage of these modalities. Overall there was a trend towards increasing TRL achieved over time in novel technology categories, although this was by no means a perfect correlation and reflects the non-linearity of development of any technology. There appears to have been a plateau in development in the last few years, limiting translation into clinical practice.

## 4 Discussion

This systematic scoping review has shown that although the monitoring of NACT response is an active and relevant field of research with 47.5 % of studies published in the last 5 years, most groups are looking to measure and/or improve the accuracy of already established methods. There are far fewer studies addressing novel technologies, and these few are at an early developmental stage as demonstrated by the low TRL.

Nevertheless, some of these studies report promising figures for accuracy of diagnosing pCR. In elastography, Evans et al. [153] measured interim tumour stiffness of 64 patients and predicted pCR with a diagnostic accuracy of 83 %. Cochran et al. [135] performed diffuse optic spectroscopy on 33 patients enrolled in ACRIN-6691, and found that tissue saturation within 10 days of commencing NACT predicted pCR with AUC = 0.92. Dameri et al. [94] calculated a cell-free DNA integrity index (DII) in 64 patients and found this predicted pCR with accuracy 82 %. MRI accuracy in the same cohort was 77 %, and by combining MRI with DII they achieved accuracy of 92.6 %. These are all relatively small studies that require validation in larger populations, but these early figures are comparative to the best reported MRI accuracies [43,44]. To achieve these accuracy levels at such an early developmental stage is encouraging, and warrants further investigation.

There was some variation among the included studies in the intended use of the technology. Some were testing prior to NACT, and aiming to predict pCR, while others were testing after NACT but before surgery, and aiming to diagnose pCR. Still others were testing at interim points during NACT, with an aim of monitoring tumour response. The distinction between technologies that predict versus diagnose pCR has a bearing on their clinical utility; the former could potentially be used in NACT planning and decision making, but to alter surgical plan (whether by de-escalating or omitting surgery altogether) would likely require a diagnostic test. Technologies aimed at monitoring the response to NACT would be better suited to tailoring of chemotherapeutic regime, and would need separate validation to be used as a diagnostic tool. Given that the aim of this scoping review is to identify the novel technologies being investigated in this field, rather than to analyse their accuracy per se, the authors felt that all of these research questions were relevant and they were all included. However in the future the three research aims ought to be developed and evaluated independently, even if it seems possible that one type of technology could potentially perform well in multiple of these roles.

### 4.1 Technology Readiness Levels

To assess the developmental stages of these technologies this scoping review has utilised Technology Readiness Levels (TRLs). The TRL framework is a globally recognised, quantitative measure that has been adopted across a range of fields, despite originating in space technology, which allows easy recognition and comparison. There are nine criteria-defined TRLs, with TRL 1 being the least developed and furthest from real-world adoption, and TRL 9 being fully implemented. Various fields have tailored the framework to their own discipline, publishing definitions for each level that reflect industry-specific development standards [72]. TRLs have for example been used to determine eligibility for funding across a range of fields supported by UK Research and Innovation including bioscience and medicine. The framework is endorsed by EURAXESS (a European Commission body) who specifically recommend it for defining progress through the drug discovery pipeline, and is also defined by the United States U.S. Department of Health & Human Services, in the context of medical countermeasures [71]. Having 9 different levels allows for fine granularity of assessment while retaining applicability across different study designs and types of technology, allowing for one system to evaluate both clinical and laboratory-based tests. Therefore assigning each paper in this scoping review that concerned novel technologies a TRL allows easy identification of how close they are to clinical adoption.

### 4.2 Novel technologies

In aiming to identify novel technologies a decision around which categories were truly novel had to be made. The modalities that are part of standard practice for monitoring of NACT in breast cancer patients in the UK (clinical breast examination, B-mode ultrasound, mammography, MRI with a contrast agent or diffusion weighted MRI) are easily excluded. Other categories in this review such as gene panels and clinical nomograms are not usual in this clinical context, but are well-established in the assessment of breast cancer more widely, and therefore were not felt to represent truly novel technologies.

Biochemical markers constituted a large proportion of the included studies (37 %, see Table 2). This was a heterogenous group, comprising a range of targets from those as commonly tested for as Ki-67 to inflammatory molecular signatures, circulating tumour cells and tumour infiltrating lymphocytes. The term ‘biomarker’ has been previously defined by The Biomarkers Definitions Working Group as “a characteristic that is objectively measured and evaluated as an indicator of normal biological processes, pathogenic processes, or pharmacologic responses to a therapeutic intervention” [73], which would encompass the targets of nearly every technology in this scoping review. Therefore for the purposes of this paper, a ‘pathological biomarker’ has been defined largely by exclusion of firstly those measurements taken via an imaging modality; secondly a combined measurement comprising a panel of genes or a set of clinico-epidemiological characteristics (clinical nomogram); thirdly any circulating nucleic acids. The latter was reported in a separate category on the basis that it constitutes a newer technological innovation, with cell-free DNA only being conclusively linked to cancer cells in 1989, and first suggested to indicate residual disease in 2008 [[74], [75], [76]] (as opposed to circulating tumour cells, which were first identified more than 150 years ago [77], and whose detection assay CellSearch received FDA approval in 2004 for use in predicting outcomes in metastatic breast cancer [78]).

The biomarker studies could be differentiated by the timing of their measurements. Most (429/493) measured their marker of interest only prior to NACT commencement, and therefore used it to predict response to NACT rather than identify response. Some (18/493) took multiple measures of the biomarker, at different timepoints during the NACT cycles, and thus had the potential to identify early tumour response, and use this to alter therapy regimes accordingly. Still others (46/493) measured their respective markers at the end of NACT, immediately prior to surgery, with a view to diagnosing tumour response, and potentially altering surgical plan. The second approach, measuring interim biomarkers, was felt to be the most clinically relevant method as an accurate monitoring technique of this kind would allow tailoring of both medical and surgical therapies to a patient's individual response, maximising the benefits of such a strategy. However, despite the novel intention in these papers, the markers themselves were not felt to be truly novel; one study measured vitamin D levels, another quantified HER2 positivity, a third used lymphocyte count (see Supplementary Fig. 1).

#### 4.2.1 Circulating nucleic acids

Of 49 studies identified investigating circulating nucleic acids, the highest AUC was reported by Liu et al., in 2023 [82]. This study recruited 243 patients, and used an approach that combined genetic profiling of the pre-NACT tumour biopsy with evaluation of circulating tumour DNA from blood samples taken at interim timepoints during NACT. The group's prediction model achieved AUC of 0.961.

There is data from as early as 2004 demonstrating a significant difference between circulating DNA concentrations in patients with breast cancer versus healthy controls [70], and from 2017 establishing the association between CNAs and longer-term prognosis following NACT for TNBC [52], but it is only more recently that circulating tumour DNA has been shown to correlate with non-response to NACT [53]. Circulating microRNAs have been developed along a similar timeline, with evidence from 2018 that an miRNA signature in exosomes could predict pCR to NACT [54]. The major direction of research in this field is divided between identifying the CNA or combination of CNAs that are the most predictive and prognostic, and developing CNA quantification and analysis from body fluids other than blood, such as urine. From a practical standpoint, this would be a less invasive test for patients and so would be well-suited for longer-term follow-up e.g. to assess for relapse [55], however in the NACT setting where patients are having regular phlebotomy, blood markers may be equally as practical. Likewise, one major advantage of CNAs is that they reflect overall tumour burden, both primary tumour and any metastases [52], as opposed to techniques like elastography which will only assess the site of the primary tumour; again, this advantage is likely to be felt most in longer-term monitoring for recurrence than in diagnosing pCR to NACT.

#### 4.2.2 Elastography

There were 15 studies investigating elastography. The highest AUC reported was by Ma et al., 2020, who studied 43 patients with HER2-negative tumours, and found tumour stiffness after the fourth cycle of NACT was predictive of pCR with an AUC of 0.960 [159].

Early evidence of elastography in breast cancer was published in 2012, where stiffness was correlated with larger invasive size and higher grade tumours [56]^,^ and decreased likelihood of pCR after NACT [57]. There are two main techniques of ultrasound elastography, shear wave elastography and strain elastography, with no significant difference between the two in terms of predicting response to NACT [59]^.^ Both techniques share the major advantage of being non-invasive and avoiding contrast, and can be easily addended to a conventional B-mode ultrasound examination, with acquisition of shear wave elastography images adding 1–2 min [60]. Indeed, elastography has recently become common practice in the initial assessment of breast lesions, although not yet approved by NICE guidance (see NICE Medtech innovation briefing [MIB15]). However, it shares similar limitations with conventional ultrasound, namely user-dependence, both in terms of selection of the region of interest [61] and delivery of the external stimulus in the case of strain elastography [62]. There is also the issue of signal attenuation caused by subcutaneous fat potentially affecting results for patients with more posterior lesions [63].

#### 4.2.3 Diffuse optical spectroscopy imaging

This scoping review identified 17 studies investigating DOS, with the highest AUC reported by Zhu et al., in 2018. The group used percentage change in tumour vascularity (as measured by total haemoglobin on near infrared imager coupled with US) to predict pCR with an AUC of 0.96 in HER2-positive tumours and 0.95 irrespective of subtype, although this was a relatively small study of 22 patients.

The first published record of diffuse optics being used in the monitoring of breast cancer response to NACT was a single case study in 2004 [64], and twenty years later the technology itself is still being actively developed. Diffuse optical methods use the scattering and absorption of near-infrared light to characterise tissue composition, and either generate images (diffuse optical imaging, using narrow spectral range but very many light source-detectors) or spectra (diffuse optical spectroscopy, using just one or two source-detectors and broadband light). Where traditional DOS/I was only able to distinguish the level of oxygenation of a tissue, by expanding the spectral range used this modality is now able to additionally characterise tissue lipid, water and collagen composition [65]. It's a non-invasive, bedside test using a handheld probe much like ultrasound, and has the advantage of being unaffected by density of breast tissue [66]. DOS delivers quantitative outputs in the form of tissue concentrations of Hb, deoxygenated Hb, lipid etc, which may mean it's less user-dependent than other forms of imaging, however the generation of these outputs requires substantial data processing and therefore is not considered ‘real-time’.

### 4.3 Clinical relevance of pCR

One thing almost every study had in common was using pathological response as their endpoint, whether they used Residual Cancer Burden (RCB) score or pCR. Pathological examination is the current gold standard for diagnosing degree of tumour response, and is a common endpoint in neoadjuvant therapy trials. However, it is not 100 % accurate at detecting residual cancer as it uses a sampling technique; pathologists cannot examine every cell, and there will unavoidably be methodological differences between different laboratories [73]. In addition, the relationship between pCR and disease-free survival (DFS) differs between cancer subtypes, with the strongest relationship observed for TNBC; Luminal A cancers have low rates of pCR but favourable prognosis, while TNBCs have high rates of pCR but poorer DFS [51]. If pCR is only prognostic for certain subtypes, then technology aiming to diagnose pCR should also be subtype-targeted. Many of the studies included here were subtype specific, and some identified that their technology's accuracy differed for different subtypes, but some papers pooled cancer types together and this may have impacted their results.

There is also a question of whether there is any benefit to seeking pCR in those cancers with poor relationship between pCR and survival, such as luminal A cancers [51]. These cancers are less likely to be offered neoadjuvant chemotherapy than more aggressive subtypes, and the aim may be to downstage and therefore de-escalate surgery rather than to cure. In this case, a test to assess tumour response rather than look specifically for pCR may be more relevant.

### 4.4 Limitations

It is the nature of any search strategy that aims to categorise a broad range of qualitative data points that granularity will be lost; in separating the papers identified into technology categories, abstraction was required and therefore it is possible that some novel technologies were lost into larger categories. However, it is only by categorising technologies broadly that we have been able to describe the large volume of work ongoing in the field.

Another challenge related to the breadth of topics covered by the papers included was how to define each technology readiness level for the purposes of this scoping review to compare across categories. This was done by consulting a number of published definitions of TRLs, particularly in biotechnology and diagnostics [67–9], and developing a consensus agreement between this paper's authors which may represent a limitation of this scoping review, as internal consistency may be better maintained than external consistency.

## 5 Conclusion

This scoping review has demonstrated that while research into the improved monitoring of NACT and diagnosis of pCR is active, more work is urgently needed to allow tailoring of regimes and de-escalation of surgery. In particular, 94 % (1248/1329) of published studies were on established technologies, with only 6 % (81/1329) on developing novel methods. It may be that established technologies will have a ceiling of diagnostic accuracy that will not progress breast cancer treatments towards more accurate de-escalation of therapy in NACT. These early-stage emerging technologies are already demonstrating promising results, and warrant further investigation and could be the key to unlocking the potential of precision oncology and surgery in breast cancer care.

## CRediT authorship contribution statement

**Georgia C. Wright:** Writing – original draft, Visualization, Resources, Investigation. **Luke T. Glover:** Writing – review & editing, Investigation. **Thomas J.E. Hubbard:** Writing – review & editing, Supervision, Methodology, Conceptualization.

## Declaration of competing interest

The authors declare that they have no known competing financial interests or personal relationships that could have appeared to influence the work reported in this paper.
