# Supplementary material for: Emerging technologies for monitoring breast cancer response to neoadjuvant chemotherapy: a systematic scoping review
Source: Breast. 2025 Dec 1;85:104663. doi: 10.1016/j.breast.2025.104663 (PMC12723055; doi:10.1016/j.breast.2025.104663)
Supplement: Multimedia component 1 [file mmc1.docx]

# Supplementary material

## Search strategy

**Literature search performed 26/1/24**

**Ovid Medline**

1. breast neoplasm* or breast cancer* or breast tumour* or breast tumor* or mammary neoplasm* or mammary cancer* or breast carcinoma* or mammary carcinoma*

2. (neoadjuvant NEAR/3 chemo*) OR ("NACT" OR "NAC")

3. ("pathological complete response" OR "PCR")

4. Predict* OR Monitor* OR assess

5. 1 AND 2 AND 3 AND 4

6. limit 5 to yr="2002 -Current"

**932 results, all imported**

**Ovid Embase**

1. breast neoplasm* or breast cancer* or breast tumour* or breast tumor* or mammary neoplasm* or mammary cancer* or breast carcinoma* or mammary carcinoma*

2. (neoadjuvant NEAR/3 chemo*) OR ("NACT" OR "NAC")

3. ("pathological complete response" OR "PCR")

4. Predict* OR Monitor* OR assess

5. 1 AND 2 AND 3 AND 4

6. limit 5 to yr="2002 -Current"

**1894 results, all imported**

**PubMed**

1. breast neoplasm* or breast cancer* or breast tumour* or breast tumor* or mammary neoplasm* or mammary cancer* or breast carcinoma* or mammary carcinoma*

2. (neoadjuvant near chemo*):ti,ab,kw OR ("NACT" OR "NAC")

3. ("pathological complete response" OR "PCR")

4. Predict* OR Monitor* OR assess

5. 1 AND 2 AND 3 AND 4

6. limit 5 to yr="2002 -Current"

**1030 results, all imported**

**Cochrane**

breast neoplasm* or breast cancer* or breast tumour* or breast tumor* or mammary neoplasm* or mammary cancer* or breast carcinoma* or mammary carcinoma* in Title Abstract Keyword AND (neoadjuvant near chemo*) OR ("NACT" OR "NAC") in Title Abstract Keyword AND ("pathological complete response" OR "PCR") in Title Abstract Keyword AND Predict* OR Monitor* OR assess in Title Abstract Keyword - with Cochrane Library publication date Between Jan 2002 and Feb 2024 (Word variations have been searched)

**3 reviews, 641 trials**

**All imported**

**Total = 4500 articles**


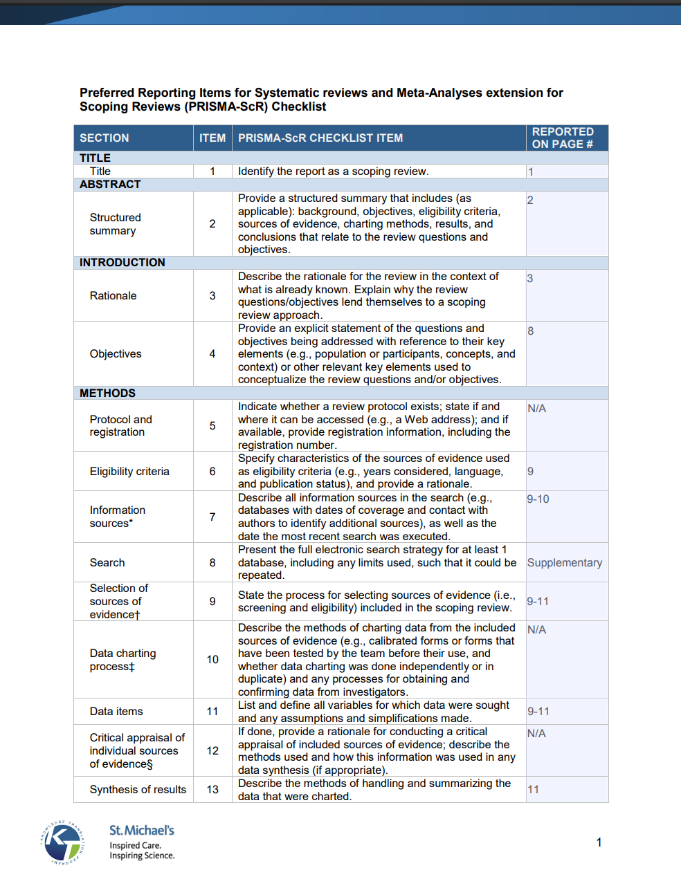

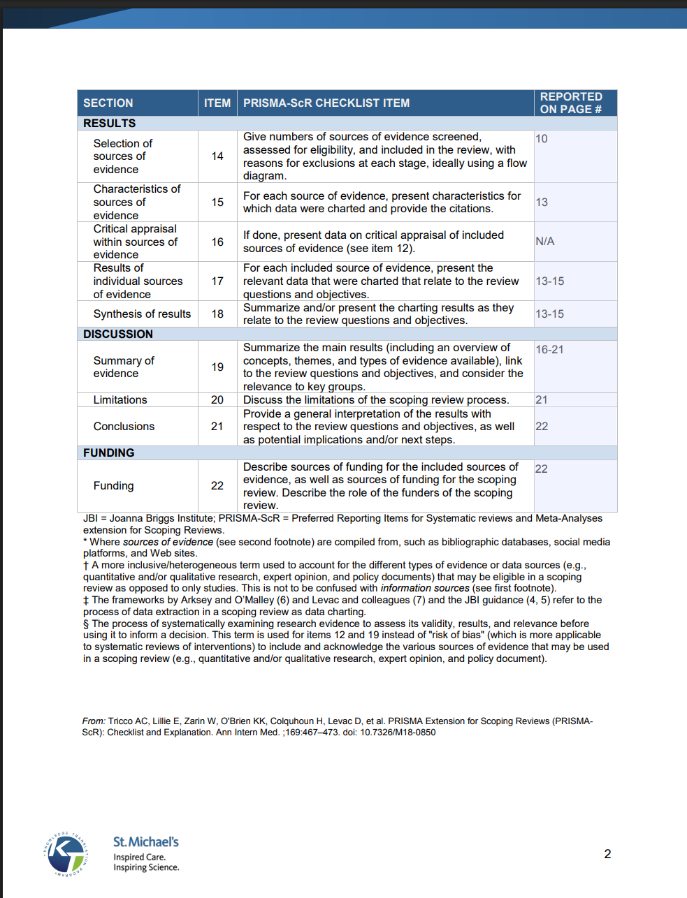


Supplementary Fig 1. PRISMA-ScR checklist


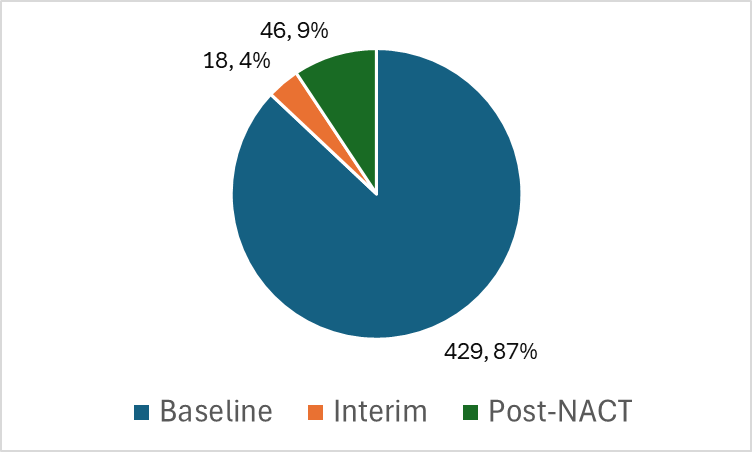


A


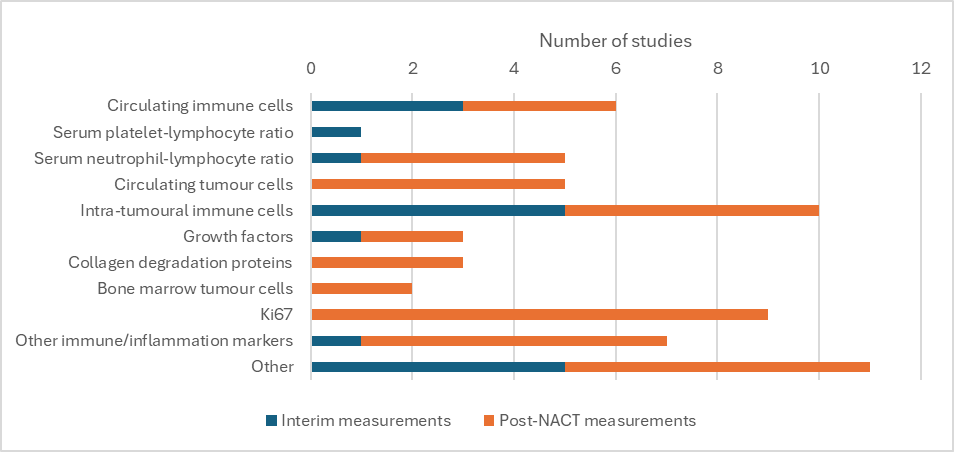


B

Supplementary Fig 2. A) Percentage of biomarker studies investigating the marker at baseline, at interim timepoints during NACT, and after completion of NACT B) Number of interim and post-NACT biomarker studies looking at each broad type of biomarker. ‘Other’ included vitamin D, gut microbiome, erythrocyte macrocytosis, SPAG4, AAGAB, N cadherin, circulating endothelial progenitor cells, serum sialic acid, PTPRO methylation, tumour lactate dehydrogenase, and a number of known oncogenes and tumour suppressor genes.
